# Integrating equity and justice in rheumatic heart disease policy: addressing reproductive health challenges

**DOI:** 10.3389/ijph.2026.1609283

**Published:** 2026-06-04

**Authors:** Panduleni Penipawa Shimanda, Tekla Shipahu Natangwe Shiindi-Mbidi

**Affiliations:** 1 Department of Nursing and Midwifery, International University of Management, Windhoek, Namibia; 2 Clara Barton School of Nursing, Welwitchia University, Windhoek, Namibia

**Keywords:** equity, policy, reproductive health, RHD, rheumatic heart disease

## Abstract

Rheumatic Heart Disease (RHD), a preventable disease of poverty, disproportionately affects women of reproductive age, highlighting a crisis at the intersection of cardiac and reproductive health. Despite effective interventions, RHD remains neglected in policy priorities. This paper uses the case of reproductive health dilemmas to demonstrate the need for the application of equity frameworks. We analyse how consequentialist, communitarian, and feminist principles can guide policymakers toward more effective and just interventions. The paper proposes a community-based model, justified through this ethical lens, and calls for policies prioritising equity to finally address RHD’s preventable burden.

## Introduction

Rheumatic heart disease (RHD) is a life-threatening condition that evolves from inadequately treated group-A streptococcal (GAS) throat infection. Antibodies that attack the original infection can also damage connective tissues in the heart, joints and skin, leading to acute rheumatic fever and, with repeated episodes, permanent valvular damage [1]. Severe RHD manifests as heart failure, atrial fibrillation, progressive disability, and premature mortality [2].

Globally, more than 46 million people were estimated to be living with RHD in 2022, and over 390,000 people die from the disease annually [3]. Labelled “a disease of poverty,” the burden is concentrated in socio-economically disadvantaged settings because overcrowding, poor sanitation and inequitable access to healthcare amplify GAS transmission [4].

However, RHD is not merely a cardiac condition; it is a profound reproductive health issue. Approximately 1.25 million new cases occur annually among women of childbearing age were estimated annually in 2021 [5]. Pregnancy can precipitate heart failure in women with RHD, and lifelong anticoagulation therapy, essential after valve surgery, poses significant risks to both mother and foetus if not meticulously managed [6, 7]. These intersecting risks underscore the inadequacy of a purely clinical approach and highlight the urgent need to integrate RHD care into sexual and reproductive health services [7].

The disease is preventable through multi-modal interventions such as reducing risk factors through improved living conditions, equitable access to healthcare, and antibiotic prophylaxis [8]. Early diagnosis and antibiotic treatment of GAS, together with improved primary healthcare, are important in preventing RHD and have been key factors in its near-eradication in high-income countries. Surgical management to repair or replace the damaged valves is a vital solution for severe disease. Lifelong post-surgical treatment includes anticoagulation, and Warfarin is commonly used for patients with mechanical prostheses. Anticoagulants are a major contributor to adverse maternal, foetal, and neonatal outcomes [9]. Although such implications can be mitigated through proper medical and obstetric management, social challenges related to RHD and access to care limits adherence to care plans [10].

This paper argues that effective RHD policy must be guided by explicit ethical frameworks to address the associated complex challenges. We examine the reproductive health dilemmas faced by women with RHD through the complementary lenses of consequentialism, communitarianism, and feminist ethics. Through synthesising these perspectives, we propose and justify a community-based, multi-level intervention designed to improve health outcomes, respect cultural contexts, and empower women. Ultimately, we advocate for policy decisions that move beyond cost-effectiveness alone to embrace equity and justice as foundational principles in the fight against RHD.

This paper is primarily intended to inform policy and programme design in low-and middle-income countries where RHD remains endemic, particularly in sub-Saharan Africa and other settings characterised by constrained health systems, gender inequities, and persistent barriers to care. The analysis speaks most directly to policy decisions concerning the integration of RHD services into primary healthcare and sexual and reproductive health programmes, as well as to the design of community-level interventions that address both clinical and social determinants of reproductive risk.

This conceptual paper is based on a narrative synthesis of interdisciplinary literature. Relevant literature was identified through targeted searches of PubMed, Scopus, and Google Scholar using combinations of key terms: “rheumatic heart disease,” “reproductive health,” “equity,” “justice,” “ethics,” “intervention,” and related concepts. Priority was given to peer-reviewed articles that could inform both the empirical basis of the reproductive health problem and the ethical justification for policy action. While this was not a systematic review, the approach was intended to support transparent and policy-relevant conceptual synthesis across diverse evidence sources.

## The intersection of RHD, gender inequality, and reproductive health

A critical barrier to care is rooted in the power dynamics that govern reproductive decision-making. In many settings where RHD is endemic, male partners, in-laws, and other family members exert significant influence over a woman’s reproductive choices [11, 12]. Women with RHD may be abandoned due to perceived infertility, while cultural pressures to have multiple children or male heirs persist [10, 11]. Thus, women face a stark choice: risk their health with pregnancy or face social and economic exclusion.

This conflict can be understood through a communitarian lens, where cultural values prioritise the community’s interests, including viewing children as economic assets and linking masculinity to fertility [13–15]. Within this framework, a man’s influence over a woman’s reproductive choices is not necessarily perceived as an injustice but as an alignment with communal goals. Policy interventions that fail to engage with these deeply held values are destined for poor uptake and resistance.

## An equity framework for ethical RHD policy

### Consequentialism

This principle, particularly utilitarianism, evaluates actions based on their outcomes, emphasizing the maximization of overall health benefits [16, 17]. From this perspective, policy may prioritise interventions that deliver the greatest good for the greatest number. For RHD, this would strongly support scaling up primary prevention (e.g., treating streptococcal sore throats) and secondary prophylaxis with penicillin, both of which are highly cost-effective, over investing in tertiary care like local cardiac surgery units.

### Communitarianism

This ethic stresses that policies must align with, and not dismiss, community values and social norms [18]. Programmes that disregard the economic significance of children or the social importance of fertility are unlikely to succeed. Instead, successful models, such as those in Indigenous Australian communities, demonstrate that engaging community leaders and employing culturally resonant approaches, like Aboriginal health workers, build trust and improve participation in RHD care [19].

### Feminist ethics

This framework highlights gender inequalities, oppression, and justice [20, 21]. It calls for policies that actively empower women, address power imbalances, and challenge harmful gender norms. A feminist approach aligns with reproductive justice, which upholds the right to have children, not have children, and parent in safe environments [22, 23]. Interventions must therefore ensure women can make informed and autonomous reproductive choices.

While this analysis focuses on consequentialist, communitarian, and feminist perspectives, other ethical frameworks, including deontological ethics may also provide important insights for RHD policy. The selection of these three frameworks reflects their relevance to public health priority setting, social embeddedness, and gender justice. However, this choice may introduce a degree of conceptual selectivity, and future work could usefully broaden the analysis to incorporate additional ethical traditions.

## Proposed intervention: multidisciplinary community-based couples and family reproductive health counselling/education programme

Translating these principles into practice, we propose a community-based couples and family counselling and education programme. This initiative is grounded in evidence demonstrating that multilevel interventions improve reproductive health outcomes.

### Model and evidence

The programme would integrate successful elements from existing models: the Male Motivator project in Malawi, which increased contraceptive uptake by improving couple communication [24]; participatory pre-marital counselling that boosted reproductive health literacy success [25, 26]; and Indigenous Australian “yarning” sessions that used community workers to deliver culturally safe RHD education quality [19]. In addition, co-designed screening initiatives in communities used task-sharing between local healthcare workers and remote cardiologists to integrate RHD screening into routine services [27]. Taken together, these examples provide a rationale for combining community engagement, couple-centred counselling, and capacity-building in RHD-related reproductive health programming. However, several of these intervention components derive from broader reproductive health or community participation literature rather than RHD-specific trials. Their application to RHD policy should therefore be understood as a reasoned extrapolation that requires context-specific testing and validation.

### Operationalisation

A multidisciplinary team, including community health workers, nurses, midwives, and peer educators, would deliver sessions in schools, community centres, and homes. The curriculum would cover RHD prevention, family planning, and reproductive rights, tailored to local contexts. Delivery could include group education sessions, facilitated couple or family dialogues, and individual follow-up for women at higher clinical or social risk. Monitoring would track prophylaxis adherence, contraceptive use, and empowerment indicators.

### Evaluation framework

A minimum evaluation framework should accompany early implementation. In the short term, approximately 12–24 months, programme indicators could include participation rates in counselling sessions, retention in follow-up, uptake of family planning counselling, changes in reproductive health knowledge, reported involvement in shared decision-making, and adherence to secondary prophylaxis. Data sources could include routine health facility records, programme monitoring tools, attendance registers, and structured participant surveys.

In the medium to longer term, approximately 3–5 years, relevant outcomes could include continuity of care, planned pregnancy counselling uptake, reduction in preventable maternal cardiac complications, and improvement in pregnancy-related outcomes among women living with RHD. Qualitative assessment would also be important to examine acceptability, changes in gender norms, women’s perceived autonomy, and the extent to which community engagement mechanisms support sustained participation.

## Alignment with equity principles

### Consequentialist

The programme prioritises highly cost-effective primary and secondary RHD prevention while reducing costly obstetric complications.

### Communitarian

The programme actively involves community advisory boards, respects cultural values in its messaging, and works within communal decision-making structures.

### Feminist

The programme creates safe spaces for women, provides gender-sensitivity training for providers, and involves men to challenge harmful norms and support equitable decision-making.

## Synthesis and policy justification through an ethical lens

While a health facility-based approach may appear less resource-intensive, its effectiveness may be limited in settings characterised by high loss to follow-up, transport barriers, low continuity of care, and unequal household decision-making power. Community-based approaches have shown promise in improving engagement in comparative evidence in RHD-specific reproductive health programmes remains limited. Accordingly, the proposed model should be interpreted as a context-sensitive policy approach rather than a universally proven solution.

Through the consequentialist lens, the “greatest good” is not achieved if interventions fail to reach those most at risk. The proposed programme’s potential to prevent maternal death, improve child health, and empower women may generate substantial and social benefits that justify the investment. The principle of “equal consideration” demands that women living with RHD in vulnerable settings be prioritised rather than overlooked because they are difficult to reach [16].

Through the communitarian lens, ignoring cultural values is a recipe for policy failure. By designing interventions that reframe the dilemma, presenting women’s health as a prerequisite for family and community wellbeing, policymakers may foster support and improve uptake. This approach recognises community strengths rather than merely pathologizing communal norms [28].

Through the feminist lens, an intervention that only engages only the patient in isolation may inadvertently leave underlying power inequalities intact. Empowering women through education, while also engaging men and family structures, is important for advancing reproductive justice and more sustainable health outcomes [20, 29, 30].

Importantly, these ethical frameworks do not always align. A communitarian emphasis on collective values and social expectations around fertility may conflict with feminist commitments to autonomy and reproductive rights. Similarly, a narrowly consequentialist focus on cost-effectiveness may undervalue interventions aimed at empowerment, relational agency, and long-term social transformation. Recognising and negotiating these tensions is essential for ethically robust RHD policy, especially where community norms and women’s interests do not neatly converge.

The transferability of this model also requires caution. Elements drawn from Indigenous Australian contexts, Malawian family planning interventions, or other setting-specific experiences cannot be assumed to translate seamlessly across all RHD-endemic regions. Adaptation to local gender norms, kinship structures, health system capacity, and community engagement traditions would be necessary in all settings. The proposed model should therefore be regarded as a flexible and adaptable framework.

## Conclusion and call to action

RHD is a biological disease intensified by poverty and inequity. Addressing its reproductive health impact requires policies grounded not only in clinical and economic evidence, but also in ethical commitment.

This paper demonstrates that integrating consequentialist, communitarian, and feminist principles offers a significant framework for equitable and effective RHD interventions. We urge policymakers to move beyond siloed approaches and support multidisciplinary, community-based programmes that integrate RHD care with sexual and reproductive health services. This can help ensure RHD policies are not only efficient, but also just, culturally responsive, and transformative for affected women and communities. Ultimately, resource allocation should affirm the value of every life and advance long-term justice in global health.

Our study has several limitations. It is based on a narrative synthesis rather than a systematic review. Some programme elements are extrapolated from adjacent evidence rather than directly tested in RHD-specific reproductive health interventions. Further, implementation research is needed to evaluate effectiveness, feasibility, equity impact, and transferability across different endemic settings.
